# Hospitals and Suicidal Patients

**Published:** 1907-11-23

**Authors:** 


### Hospitals and Suicidal Patients.

An interesting question is at present under consideration at one of the London general hospitals—namely, What is to be done in cases of attempted suicide which are admitted into the hospital for treatment? Are the police authorities to be informed? From the administrative point of view the matter is purely financial. Whenever such a case is admitted it is obviously essential that a special attendant should be provided by day and by night to prevent a repetition of the attempt. If the patient is brought in by the police, the police authorities will undertake this responsibility and provide the attendants. If the patient's attempt at suicide does not come under the notice of the police, the hospital authorities must either provide and pay the attendants, or must notify the police authority. A wealthy hospital would provide the special attendants at its own expense. Unfortunately, few hospitals can ungrudgingly bear these charges.

The ethical point affects both the resident medical officers and the administrative body. It is well recognised in the medical profession that a patient's confidence is sacred, and that matters which come to the knowledge of the doctor, in the course of his treatment of the case, must not be divulged, except under the order of a judge in court or for the prevention of crime. The doctor should do nothing which would tend to prevent the individual from seeking medical advice, and, above all, must not take such a view of his position as to lead him to act as an agent for the punishment of criminal offences. Such being the case, the medical officer of a hospital is in a very difficult position if the committee of management pass a rule to the effect that the police should be informed in all cases of attempted suicide which are admitted to the hospital. He is the servant of the hospital, for he is boarded free of charge, and generally receives a more or less inadequate salary. An analogous position is that of the doctor who is employed by a lady to examine her maidservant and finds her pregnant. The doctor is the agent or servant of the lady, and is paid his fees by her; yet it has been held that, without the maid's permission, he must not divulge her secret to the mistress. From the point of view of the committee of management of the hospital, it may be urged that the secretary, being directly responsible for the management of the hospital, has the right to be informed of the nature of the illness of each patient admitted. No secret is made of the ailments of hospital patients. But it must not be forgotten that, though hospital patients may have no objection to their ailments being more or less public property in the hospital itself, it is a very different matter to publish the information to a public and punitive body, such as the police authorities. The hospital might become involved in an action for damages, based on breach of confidence.

The crime of attempted suicide is not regarded by the law with severity. Usually the discomfort, actual pain, and publicity attached thereto are regarded as quite sufficient punishment, and, if the case comes before a court, the patient is merely handed over to some responsible friend or relative, with a few words of advice or warning. Many suicidal patients are definitely ill, mentally or physically, and require medical treatment rather than the rigours of the police cell. Sometimes attempts at suicide, occasionally successful, are made by patients actually under treatment for acute illness. It is doubtful whether the police authorities would recognise any responsibility in such cases.

It is obvious, therefore, that the resident medical officers should regard themselves as ethically bound not to report such cases to the police, but that, if the committee so desire, they may be justified in informing the secretary, who would then act in accordance with the instructions of his committee. Comparatively few suicidal patients are admitted to any one hospital in the course of the year, and the expense of their supervision is no great item. We are of the opinion that it is both ethically and politically sound for the hospital to bear this expenditure rather than to incur the odium of being regarded in the eyes of the public as police agents or common informers. Notification would no doubt reduce the expenditure of the particular hospital by driving these patients to one which took a more charitable and, we might add, more Christian view of its duty and responsibility to the unfortunate sufferer claiming its assistance.

